# Higher serum 25(OH)D level is associated with decreased risk of impairment of glucose homeostasis: data from Southwest China

**DOI:** 10.1186/s12902-018-0252-4

**Published:** 2018-05-09

**Authors:** Danting Li, Haoche Wei, Hongmei Xue, Jieyi Zhang, Mengxue Chen, Yunhui Gong, Guo Cheng

**Affiliations:** 1 Department of Nutrition, Food Safety and Toxicology, West China School of Public Health, Sichuan University, No.16, Section 3, Renmin Nan Road, Chengdu, 610041 Sichuan China; 2 Center of Growth, Metabolism and Aging, Collage of Life Sciences, Sichuan University, Chengdu, China; 3 Department of Obstetrics and Gynecology, West China Second University Hospital, Sichuan University, Chengdu, China

**Keywords:** Vitamin D, Glucose homeostasis, Pre-diabetes, Adult

## Abstract

**Background:**

Recent epidemiological studies have suggested inverse associations between vitamin D status and metabolic diseases including type 2 diabetes (T2DM). The aim of this study was to examine whether a higher serum 25-hydroxyvitamin D (25(OH)D) was associated with a more favorable glucose homeostasis among adults without diabetes in Southwest China.

**Methods:**

Serum 25(OH)D concentration was measured in a cross-sectional sample of 1514 adults without diabetes aged 25–65 years recruited from Southwest China. Indices describing glucose homeostasis included fasting plasma glucose (FPG), fasting insulin, glycated hemoglobin (HbA_1c_), the homeostatic model assessment 2-insulin resistance (HOMA2-IR) and odds of pre-diabetes. Data were analyzed by multivariable-adjusted regression models.

**Results:**

The average serum 25(OH)D was 22.66 ng/ml, and percentages of vitamin D deficiency [25(OH)D < 20 ng/ml], insufficiency [20 ≤ 25(OH)D ≤ 30 ng/ml] were 47.6 and 32.2%, respectively. Serum 25(OH)D was inversely associated with fasting insulin (*P* = 0.0007), HbA_1c_ (*P* = 0.0001) and HOMA2-IR (*P* = 0.0007), but not with FPG, after adjusting for age, gender, monthly personal income, smoking status, energy intake, moderate-to-vigorous physical activity (MVPA) and waist circumference (WC). Compared with the lowest 25(OH)D tertile, the odds ratio for pre-diabetes in the highest tertile was 0.68 (95%CI: 0.47-0.99) after adjustment for cofounders. In the following stratified analyses according to weight status, we only observed this inverse association between serum 25(OH)D and pre-diabetes in overweight or obese adults (*n* = 629, *P* = 0.047), but not in their counterparts with BMI < 24 kg/m^2^.

**Conclusions:**

Our results advocate that a higher serum 25(OH)D level is associated with decreased risk of impairment of glucose homeostasis among adults without diabetes in Southwest China. Further studies are warranted to determine the role of vitamin D in glucose homeostasis.

**Electronic supplementary material:**

The online version of this article (10.1186/s12902-018-0252-4) contains supplementary material, which is available to authorized users.

## Background

Over the past decades, the prevalence of type 2 diabetes (T2DM) among Chinese adults has increased from 2.5% in 1994 [1] to 11.6% in 2013 [2]. Additionally, almost half of the adult population had pre-diabetes [2], a major risk factor for the development of T2DM [3].

It is becoming clear that vitamin D status is related to cancer [4], multiple sclerosis [5], cardiovascular disease [6], and diabetes [7–9], besides its role in the modulation of calcium absorption and bone metabolism. Moreover, Vitamin D deficiency has now recognized as a worldwide concern [10]. Zhen et al. [11]. reported that northwest Chinese adults exhibit high prevalence (75.2%) of vitamin D deficiency [25(OH)D < 20 ng/mL].

The associations between vitamin D and factors involved in glucose homeostasis have drawn a great deal of attention recently. 25-hydroxyvitamin D (25(OH)D), the sum of both 25(OH)D2 and 25(OH)D3, is a generally accepted biomarker of vitamin D status. Several observational studies have reported that serum 25(OH)D is negatively associated with fasting plasma glucose (FPG) and insulin among western populations [12–15]. In addition, patients with T2DM had lower serum 25(OH)D compared to control subjects without diabetes [16]. Notably, vitamin D metabolism and its nutritional status were found to differ by ethnicity [8], nonetheless evidence from Asian populations is limited. Available data from China report that 25(OH)D is negatively associated with insulin resistance in patients with T2DM [17], while studies conducted among participants without diabetes are much less [18]. Besides, existing studies mainly focus on the relation of vitamin D with partial indicators (e.g. FPG or insulin) which can not cover the general status of glucose homeostasis.

Therefore, using data from a representative study among Chinese adults without diabetes, we investigated whether a higher serum 25(OH)D was associated with a more favorable glucose homeostasis (a. relative lower level of FPG, fasting insulin, HbA_1c_ and HOMA2-IR within their normal ranges; b. lower odds of pre-diabetes). Furthermore, our results may highlight the importance of improving vitamin D status in the general population.

## Methods

### Study population

We used data from an ongoing population-based prospective study conducted in Southwest China initiated in September 2013, which aimed to investigate the health impact of nutritional and lifestyle factors on the development of several chronic diseases, as described elsewhere [19]. Using a cluster random sampling design stratified by urban and rural locations, a representative sample of civilian aged 25–65 years was recruited from the general population in Chengdu, Southwest China. The participants were invited to the study center for interviews. Generally, each visit included anthropometric measurements, medical examinations, questionnaires and face-to-face interviews by trained investigators about nutrition-related behaviors, lifestyles and social status. However, the following participants were excluded from the study: a) if they had major organ diseases, including heart, liver or kidney disease; b) if they had mental diseases; c) if they were taking hormone-based drugs and other medicines that affect blood glucose and lipids; or d) if they were pregnant or lactating women. The study was approved by the Ethics Committee of Sichuan University, and all participants provided written informed consent.

For the reason that serum 25(OH)D concentration was not measured in 2013 and 2014, eligible data in the present analysis were identified from the baseline survey conducted from March to October, 2015. Participants in survey 2013-2014 did not differ in gender, age, location and educational status from those who were included in our study.

### Laboratory methods

All participants were requested to have an overnight fast of at least 10 h. Peripheral venous blood samples were centrifuged, aliquoted and stored at − 80 °C until measurement. 25(OH)D, calcium^2+^ and insulin were assayed from serum samples, while plasma samples were used to measure the concentrations of FPG. Finally, HbA_1c_ was quantified from resolved erythrocytes. Serum 25(OH)D was measured using high-performance liquid chromatography (Agilent 1260 HPLC, Shanghai, China) and the intra-assay Coefficient of Variation (CV) was less than 5%. Vitamin D nutritional status was assessed as “deficiency” (< 20 ng/ml), “insufficiency” (20-30 ng/ml) or “sufficiency” (> 30 ng/ml) [20]. Serum calcium^2+^, which is closely related to serum 25(OH)D, was measured by automatic biochemistry analyzer. Serum insulin was assayed with chemiluminescence enzyme immunoassay within 4 h, and the intra-assay CV was 2.4%. Plasma glucose was measured by hexokinase assay on blood collected into fluoridated EDTA tubes within 2 h with an intra-assay CV of 2.5%. HbA_1c_ was quantified with high-performance liquid chromatography (Bio-Rad D10 automatic analyzer, Shanghai, China) (intra-assay CV: 1.1%) at the clinical laboratory center in Chengdu, which was certified by the National Glycohemoglobin Standardization Program. Finally, the insulin resistance index (HOMA2-IR) was calculated using updated homeostasis model assessment methods (http://www.dtu.ox.ac.uk/homacalculator/) according to the Wallace formula [21].

### Anthropometric measurements

Anthropometric measurements were performed by trained medical workers according to the standard procedures [22], with the participants dressed in underwear only, barefoot, and women’s hair uncovered. Waist circumference (WC) was measured without clothes midway between the lower rib margin and iliac crest, to the nearest 0.1 cm, after inhalation and exhalation, using inelasticity tape. Height and weight were measured to the nearest 0.1 kg and 0.1 cm, respectively, with an ultrasonic meter (Weight and Height Instrument DHM-30, China). Weight, height and WC were each averaged based on two measurements. Body mass index (BMI) was calculated as weight (kg) divided by height squared (m^2^) and was categorized as underweight (BMI < 18.5 kg/m^2^), normal weight (18.5 kg/m^2^ ≤ BMI < 24 kg/m^2^), overweight (24 kg/m^2^ ≤ BMI < 28 kg/m^2^), or obese (BMI ≥ 28 kg/m^2^) using the standard of Working Group on Obesity in China [23].

### Definition of pre-diabetes

Pre-diabetes, based on glycaemic parameters above normal but below diabetes thresholds, is a high risk state for diabetes [24]. In our study, it was defined using the updated classification and diagnosis of diabetes of American Diabetes Association [3] as presentation of one or more of the following results: a) HbA_1c_ of 5.7-6.4%; b) Fasting blood glucose of 100-125 mg/dl (5.6-6.9 mmol/L).

### Other covariates

Information on socio-demographic characteristics, lifestyle, dietary intake, and other potential confounders were collected by interviewer-administered questionnaires in a face-to-face interview.

For the present analysis, we assessed socio-demographic factors potentially associated with serum 25(OH)D level and glucose homeostasis, which included gender, age (years), education level (≤ 6, 6~ 12, or > 12 years of schooling), occupation (mental worker, physical worker, retired or unemployed) [25] and monthly personal income (≤ 1800 Yuan, 1800~ 3200 Yuan, or > 3200 Yuan) [26]. We also collected data on lifestyle, including smoking status (current smokers, ex-smokers and non-smokers), sleeping and stress, and physical activities. To quantify the intensity of physical activities, the energy cost of moderate-to-vigorous physical activity (MVPA) was measured in metabolic equivalents-hours per week (MET-hours/week) [27].

Dietary data were collected on two random days within a 10-day period by trained investigators using a validated 24-h dietary recall [19]. Participants were asked to recall all foods and beverages they consumed and the corresponding timing. Dietary intake data from 24-h dietary recall were converted into energy using the continuously updated in-house nutrient database based on China Food Composition 2009 [28]. And in this analysis, total energy intake for each participant was calculated as individual means of two-day 24-h dietary recall in kcal/day. Alcohol beverage consumption (cups/d) and coffee consumption (cups/wk) were accessed by food frequency questionnaire.

In addition, season of blood drawn (spring: March to May, summer: June to August; autumn: September to November; winter: December to February) was recorded.

### Statistical analysis

All statistical analyses were performed with SAS software (SAS, version 9.3, 2011, SAS Institute Inc., Cary, NC, USA.). A *P* value < 0.05 was considered statistically significant, except for interaction tests, where *P* < 0.1 was considered significant. Normality of all continuous variables was examined using normal probability plots and the Kolmogorov-Smirnov test. Given their non-normality, all continuous variables were presented as median (25th percentile, 75th percentile). As the initial analysis indicated no interaction between gender and relations of serum 25(OH)D with FPG, HbA_1c_, insulin levels, HOMA2-IR (range of *P*-value: 0.4–0.9), we pooled the sample in the follow-up analyses.

We cross-classified the study sample into categories of tertiles (T1-T3) of serum 25(OH)D to examine the distribution of baseline parameters. We tested differences in proportions using Student t-tests for normally-distributed continuous variables, the Wilcoxon rank-sum for non-normally distributed continuous variables and the Chi-square test for categorical variables, respectively.

To investigate the associations of serum 25(OH)D with glucose homeostasis, multivariable linear generalized regression models (PROC GLM in SAS) were performed. Serum 25(OH)D was defined as the independent variable in separate models. Glucose homeostasis including fasting insulin, FPG, HbA_1c_ and HOMA2-IR were dependent variables in separate models. The independent and dependent variables that enter the linear regression models were non-normally distributed continuous variables. To improve the fitting effect of the models, log-transformed values of insulin, FPG, HbA_1c_ and HOMA2-IR were used in the models.

In the basic models, the correlation analyses between serum 25(OH)D and glucose homeostasis (insulin, FPG, HbA_1c_, HOMA2-IR) were carried out first. In a further analysis, the following variables potentially affecting these associations were added: gender, age (years), educational level (≤ 6 years, 6~ 12 years, or > 12 years of theoretical education), monthly personal income (≤ 1800 Yuan, 1800~ 3200 Yuan, or > 3200 Yuan), occupation (mental worker, physical worker, retired or unemployed), smoking status (current smokers, ex-smokers and non-smokers), MVPA (MET-hour/week), total energy intake (kcal/d), alcohol beverage consumption (cups/d), coffee consumption (cups/wk), serum calcium^2+^ (mmol/L), season of blood drawn (spring, summer, autumn and winter) and WC (cm) or BMI (kg/m^2^). Each variable was initially considered separately, and variables that had their own independent significant effect in the basic models or that substantially modified the association of serum 25(OH)D with each variable of glucose homeostasis were included in the multivariate analyses. Thus, age, gender, monthly personal income, smoking status and season of blood drawn were retained in model A. In a further step, we additionally adjusted for MVPA and energy intake (model B). WC was checked as a potential confounder in model C. The adjusted means were the least-squares means predicted by the model when the other variables were held at their mean values. Then the least-squares means and 95% confidence interval (95%CI) computed by the linear models were back transformed and then presented in the results.

Finally, multivariate logistic regression analyses were used to determine the association of serum 25(OH)D with the odds of pre-diabetes. To enhance comparability, models were constructed in analogy to the multivariable linear regression analyses. To explore interaction of weight status on this association, we divided the participants into two groups according to their weight status (overweight or not) and performed stratified analyses. Estimates are presented as odds ratios (ORs) with 95% CI.

## Results

**Fig. 1:**
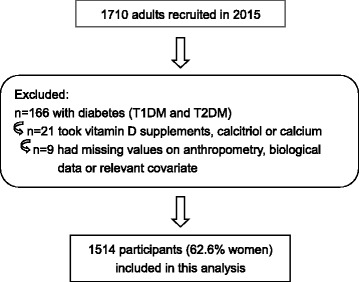
Flowchart for the study sample

A total of 1710 adults (654 men and 1056 women) had their blood drawn, completing the anthropometric measurements and questionnaires initially in 2015. We excluded individuals who had already been diagnosed with diabetes mellitus (*n* = 166) and adults who had taken vitamin D supplements, calcitriol or calcium (*n* = 21). Furthermore, participants who had missing value on anthropometric or biological data, or information on relevant covariates were excluded (*n* = 9). Therefore, this analysis was based on a final sample of 1514 participants (Fig. 1).

General characteristics of the study sample are presented in Table 1. Participants (62.6% women) included in the present analysis had a mean age of 48.74 years. The average serum 25(OH)D concentration was 22.66 ng/ml, and percentages of vitamin D deficiency and insufficiency were 47.6 and 32.2%, respectively. Almost 65.2% of the participants had pre-diabetes (Additional file 1**:** Table S1), and the prevalence of overweight or obesity was 41.5% in our study sample.

**Table 1:** Characteristics^a^ of study sample by gender (*n* = 1514)

Characteristics	Total	Male	Female
n (%)	1514 (100.0)	566 (37.4)	948 (62.6)
Age (yrs)	51.5 (37.9, 60.6)	46.7 (32.2, 60.4)	53.2 (42.1, 60.6)
Serum 25(OH) D (ng/ml)	20.7 (15.2, 27.9)	19.7 (14.6, 25.1)	21.8 (15.7, 29.9)
Pre-diabetes^b^ (n (%))	987 (65.2)	383 (67.7)	604 (63.7)
Blood parameters			
Fasting plasma glucose (mmol/L)	5.20 (5.51, 5.85)	5.58 (5.28, 5.89)	5.46 (5.16, 5.84)
Fasting insulin (μIU/mL)	6.40 (4.13, 10.13)	6.70 (3.95, 10.00)	6.30 (4.20, 10.20)
HbA_1c_ (%)	5.50 (5.20, 5.80)	5.50 (5.20, 5.80)	5.60 (5.30, 5.80)
HOMA2-IR^c^	0.87 (0.55, 1.35)	0.89 (0.52, 1.34)	0.84 (0.59, 1.37)
Serum calcium^2+^ (mmol/L)	2.42 (2.33, 2.51)	2.45 (2.36, 2.55)	2.40 (2.32, 2.50)
Anthropometric parameters			
Overweight^d^ (n (%))	629 (41.5)	288 (50.9)	341 (35.9)
Overweight^e^ (n (%))	442 (29.2)	212 (37.5)	230 (24.3)
Body mass index (kg/m^2^)	23.2 (21.0, 25.4)	24.1 (21.9, 26.1)	22.7 (20.8, 25.0)
Waist circumference (cm)	84.1 (77.5, 91.0)	86.4 (80.5, 92.4)	82.5 (76.0, 89.1)
Social-demographic data			
High education level^f^ (n (%))	676 (44.7)	311 (55.0)	365 (38.5)
High monthly personal income^g^ (n (%))	576 (38.0)	319 (56.4)	257 (27.1)
Mental worker^h^(n (%))	510 (33.7)	204 (36.0)	306 (32.3)
Lifestyles			
Smoking status (current, n (%))	217 (14.4)	192 (33.9)	25 (2.6)
MVPA (MET-hour/week)^i^	95.4 (60.6, 144.3)	78.5 (49.5, 122.1)	105.8 (69.5, 155.5)
Total energy intake (kcal/d)	1552.2 (1251.3, 1886.9)	1808.9 (1494.0, 2141.5)	1409.3 (1167.1, 1705.4)
Season of blood drawn (summer^j^, n (%))	372 (24.6)	142 (25.1)	230 (24.3)

^a^Values are median (25th percentile, 75th percentile) for non-normally-distributed continuous variables and n (%) for categorical variables

^b^Pre-diabetes was defined using the updated classification and diagnosis of diabetes of American Diabetes Association (ADA) [3]

^c^HOMA2-IR, Homeostasis model assessment 2-insulin resistance, calculated by Wallace Formula [21]

^d^Body mass index ≥24.0 kg/m^2^ [23]

^e^Body mass index ≥25.0 kg/m^2^ [48]

^f^At least 12 years of school education

^g^Monthly personal income at least ≥3200 CNY (Chinese Yuan), which is moderate level among the general population in Southwest China [26]

^h^Mental worker includes professional and technical personnel (teacher/policeman/doctor etc), legislator & administrator, businessman and student [25]

^i^MVPA: moderate-to-vigorous physical activity (MET-hour/week) [27]. MET: Metabolic equivalent

^j^Summer in Southwest China is from June to August generally

Across 25(OH)D tertiles, participants in higher serum 25(OH)D tertiles had significantly lower fasting insulin, HOMA2-IR, serum calcium^2+^ and monthly personal income, in addition, they were more likely to have their blood drawn in summer and to engage in more physical activities than those in lower tertiles of serum 25(OH)D concentration. The differences in the associations (Vitamin D and glucose homeostasis) between the three proposed groups were not statistically significant in regard to FPG or HbA_1c_ and several other variables. (Table 2).

**Table 2:** Characteristics of study sample by tertiles of serum 25(OH)D (*n* = 1514)^a^

	Tertiles of serum 25(OH)D (ng/ml)			*P*
	Tertile 1 12.6 (9.5, 15.1)^b^	Tertile 2 20.6 (18.6, 23.1)^b^	Tertile 3 31.9 (27.9, 38.9)^b^	
n (%)	504 (33.3)	505 (33.4)	505 (33.4)	–
Age (yrs)	58.9 (47.0, 64.9)	47.8 (34.9, 57.9)	57.7 (49.5, 62.2)	< 0.0001
Male (n (%))	203 (40.3)	222 (44.0)	141 (27.9)	0.0001
Pre-diabetes^c^ (n (%))	323 (64.1)	334 (66.1)	330 (65.4)	0.847
Blood parameters				
Fasting plasma glucose (mmol/L)	5.51 (5.22, 5.82)	5.51 (5.17,5.84)	5.49 (5.20, 5.90)	0.929
Fasting insulin (μIU/mL)	7.00 (4.40, 10.86)	6.50 (4.20, 10.40)	5.80 (3.90, 9.35)	0.014
HbA_1c_ (%)	5.50 (5.20, 5.80)	5.50 (5.20, 5.80)	5.60 (5.30, 5.80)	0.483
HOMA2-IR^d^	0.95 (0.59, 1.45)	0.87 (0.56, 1.39)	0.78 (0.52, 1.26)	0.018
Serum calcium^2+^ (mmol/L)	2.44 (2.36, 2.52)	2.43 (2.33, 2.51)	2.40 (2.30, 2.50)	0.006
Anthropometric parameters				
Overweight^e^ (n (%))	208 (41.2)	202 (40.1)	219 (43.3)	0.707
Overweight^f^ (n (%))	146 (29.0)	138 (27.4)	158 (31.3)	0.566
Body mass index (kg/m^2^)	22.9 (20.9, 25.3)	23.3 (21.1, 25.2)	23.4 (21.1, 25.6)	0.372
Waist circumference (cm)	83.5 (77.4, 89.8)	84.1 (77.3, 90.0)	84.7 (78.1, 92.2)	0.119
Social-demographic data				
High education level^g^ (n (%))	237 (47.0)	243 (48.1)	196 (38.8)	0.042
High monthly personal income^h^ (n (%))	201 (39.9)	197 (39.0)	178 (35.3)	0.0005
Mental worker^i^ (n (%))	188 (37.3)	196 (38.8)	126 (25.0)	< 0.0001
Lifestyles				
Smoking status (current, n (%))	72 (14.3)	79 (15.6)	66 (13.1)	0.539
MVPA (MET-hour/week)^j^	90.0 (54.6, 139.4)	89.24 (58.3, 137.4)	105.90 (66.6, 153.5)	0.019
Total energy intake (kcal/d)	1563.3 (1250.7, 1884.8)	1575.7 (1261.0, 1963.3)	1530.0 (1232.6, 1839.8)	0.361
Season^k^ (summer, n (%))	84 (16.7)	138 (27.3)	150 (29.7)	< 0.0001

^a^Values are median (25th percentile, 75th percentile) for non-normally-distributed continuous variables and n (%) for categorical variables. For non-normally distributed data, Kruskal-Wallis test was used to test the differences of the parameters among the tertiles of 25(OH)D, and for categorical variables, chi-square test were used

^b^Values are median (25th percentile, 75th percentile) in tertiles of vitamin D (ng/ml)

^c^Pre-diabetes was defined using the updated classification and diagnosis of diabetes of American Diabetes Association (ADA) [3]

^d^HOMA2-IR, Homeostasis model assessment 2-insulin resistance, calculated by Wallace Formula [21]

^e^Body mass index ≥24.0 kg/m^2^ [23]

^f^Body mass index ≥25.0 kg/m^2^ [48]

^g^At least 12 years of school education

^h^Monthly personal income at least ≥3200 CNY (Chinese Yuan), which is moderate level among the general population in Southwest China [26]

^i^Mental worker includes professional and technical personnel (teacher/policeman/doctor etc), legislator & administrator, businessman and student [25]

^j^MVPA: moderate-to-vigorous physical activity (MET-hour/week) [27]. MET: Metabolic equivalent

^k^Summer in Southwest China is from June to August generally

The associations of tertiles of Vitamin D with glucose homeostasis are shown in Table 3. Multiple linear regression analysis showed that serum 25(OH)D concentration was inversely related to fasting insulin (*P* = 0.016), HbA_1c_ (*P* = 0.0003) and HOMA2-IR (*P* = 0.016) in these non-diabetic participants after adjustment for age, gender, monthly personal income, smoking status and season of blood drawn (model A), while association between vitamin D and FPG was not statistically significant (*P* > 0.05, after adjusting for confounders mentioned above). Further adjusting for MVPA, energy intake (model B), or including additional adjustment for WC (model C) did not materially change these inverse associations. Adults in the highest serum 25(OH)D concentration tertile had 12.4% lower fasting insulin (*P* = 0.0007), 2.2% lower HbA_1c_ (*P* = 0.0001) and 12.3% lower HOMA2-IR (*P* = 0.0007) than those in the lowest tertile.

**Table 3:** Multiple linear regression least-squares means and 95% confidence interval for the association of tertiles of serum 25(OH)D (ng/ml) with glucose homeostasis (*n* = 1514)^a^

	Tertiles of serum 25(OH)D (ng/ml)			*P* for trend
	Tertile 1 12.6 (9.5, 15.1)^b^	Tertile 2 20.6 (18.6, 23.1)^b^	Tertile 3 31.9 (27.9, 38.9)^b^	
Fasting insulin (μIU/mL)				
Model A^c^	8.36 (7.59, 9.12)	8.00 (7.25, 8.75)	7.47 (6.68, 8.26)	0.016
Model B^d^	8.35 (7.58, 9.12)	7.98 (7.23, 8.73)	7.45 (6.66, 8.24)	0.015
Model C^e^	8.00 (7.31, 8.70)	7.63 (6.95, 8.31)	7.01 (6.29, 7.73)	0.0007
Fasting plasma glucose(mmol/L)				
Model A^c^	5.56 (5.48, 5.64)	5.55 (5.47, 5.63)	5.52 (5.44, 5.61)	0.331
Model B^d^	5.56 (5.48, 5.64)	5.55 (5.47, 5.63)	5.52 (5.43, 5.60)	0.291
Model C^e^	5.55 (5.47, 6.63)	5.53 (5.46, 5.61)	5.50 (5.42, 5.58)	0.197
HbA_1c_ (%)				
Model A^c^	5.57 (5.51, 5.63)	5.53 (5.46, 5.59)	5.46 (5.40, 5.52)	0.0003
Model B^d^	5.57 (5.51, 5.63)	5.52 (5.46, 5.82)	5.45 (5.39, 5.52)	0.0002
Model C^e^	5.57 (5.50, 5.63)	5.52 (5.46, 5.58)	5.45 (5.39, 5.51)	0.0001
HOMA2-IR				
Model A^c^	1.11 (1.01, 1.21)	1.06 (0.97, 1.16)	0.99 (0.89, 1.10)	0.016
Model B^d^	1.11 (1.01, 1.21)	1.06 (0.96, 1.16)	0.99 (0.89, 1.10)	0.015
Model C^e^	1.06 (0.97, 1.16)	1.02 (0.93, 1.10)	0.93 (0.84, 1.03)	0.0007

^a^Values are models least-squares means and 95% confidence interval. Linear trends (*P* for trend) were obtained with vitamin D concentrations as continuous variables;

^b^Values are median (25th percentile, 75th percentile) in tertiles of Vitamin D (ng/ml);

^c^Model A: adjusted for age, gender, monthly personal income, smoking status and season of blood drawn;

^d^Model B: additionally adjusted for MVPA and energy intake;

^e^Model C: additionally adjusted for waist circumference

Table 4 outlines the association of tertiles of Vitamin D with the odds of pre-diabetes. A significantly 32% lower odds of pre-diabetes was observed for adults in the highest tertile of serum 25(OH)D (OR: 0.68, 95%CI: 0.47-0.99) compared with those in the lowest tertile after adjustment for potential confounders. We further discovered an interaction of weight status on this association (*P* = 0.06). In stratified analyses, overweight individuals in the highest tertile of serum 25(OH)D had lowest risk of pre-diabetes (*P* = 0.047), but not in individuals with BMI < 24 kg/m^2^. To compare these results with western adults, we conducted a sensitivity analysis using the criteria of World Health Organization to define overweight and observed similar result patterns (Additional file 2**:** Table S2).

**Table 4:** Multiple logistic regression odds ratio (OR) and 95% confidence interval (CI) for the association of tertiles of serum 25(OH)D (ng/ml) with pre-diabetes^a^

	OR (95%CI)			*P* for trend
Pre-diabetes^c^ (yes or no)	Tertile 1 12.6 (9.5, 15.1)^b^	Tertile 2 20.6 (18.6, 23.1)^b^	Tertile 3 31.9 (27.9, 38.9)^b^	
Total (*n* = 1514)				
Model A^d^	1.00	1.07 (0.75, 1.52)	0.70 (0.48, 1.01)	0.056
Model B^e^	1.00	1.07 (0.74, 1.52)	0.69 (0.47, 0.99)	0.049
Model C^f^	1.00	1.06 (0.74, 1.52)	0.68 (0.47, 0.99)	0.046
BMI < 24 kg/m^2^ (*n* = 885)				
Model A^d^	1.00	1.32 (0.84, 2.08)	0.86 (0.53, 1.38)	0.182
Model B^e^	1.00	1.36 (0.87, 2.14)	0.86 (0.53, 1.39)	0.153
BMI ≥ 24 kg/m^2^ (*n* = 629)				
Model A^d^	1.00	0.68 (0.36, 1.27)	0.45 (0.24, 0.85)	0.052
Model B^e^	1.00	0.65 (0.34, 1.22)	0.45 (0.23, 0.84)	0.047

^a^Values are odds ratio and 95% confidence interval. Linear trends (*P* for trend) were obtained with vitamin D concentrations as continuous variables;

^b^Values are median (25th percentile, 75th percentile) of in tertiles of Vitamin D (ng/ml);

^c^Using the Classification and Diagnosis of diabetes of American Diabetes Association to classify pre-diabetes [3];

^d^Model A: adjusted for age, gender, average personal income per month, smoking status and season of blood drawn;

^e^Model B: additionally adjusted for MVPA and energy intake;

^f^Model C: additionally adjusted for waist circumference

## Discussion

This study suggests that a higher vitamin D level was associated with a more favorable glucose homeostasis among non-diabetic adults in Southwest China. Moreover, a poor vitamin D status was significantly related with an increased risk of pre-diabetes especially in overweight or obese adults.

As indicated in previous studies [8, 12, 13], our findings suggested inverse associations of serum 25(OH)D with fasting insulin and insulin resistance. Of note, superior than the traditional assessment of HOMA1-IR, our study used HOMA2-IR to assess insulin resistance [29, 30]. The underlying mechanisms that may explain these associations have not been well understood, many possibilities being raised: a) vitamin D appears to exert effects on pancreatic β-cells secretory function and insulin sensitivity through direct modulation of gene expression via vitamin D receptors (VDRs) or regulation of calcium influx [31, 32]; b) VDR gene polymorphisms have been recently suggested to associate with variation in insulin secretion [33, 34]; and c) insufficient vitamin D usually results in increased serum parathyroid hormone which in turn has been found to be related to impaired glucose tolerance and decreased insulin sensitivity in healthy adults [35].

Serum 25(OH)D in the present study was shown to be negatively associated with HbA_1c_, the longer-term marker of glycemic status, which was in line with a cross-sectional population-based survey from National Health and Nutrition Examination Survey (NHANES) 2003-2006 [36]. Liu et al. [12] reported an inverse association between serum 25(OH)D and FPG as well, whereas we did not observe that. One reason for the divergence might be that FPG is a short-term indicator of glycemic status susceptible to participants’ diet and emotion at specific times. Moreover, evidence has suggested the decrease in HbA_1c_ with increasing 25(OH)D was the steepest in levels < 65 nmol/L (26 ng/ml), with some small decreases with further increases [37]. In this scenario, the relative poorer vitamin D status of our study sample may lead to a more overt relation of serum 25(OH)D with HbA_1c_ rather than with FPG.

In this study, low vitamin D status was associated with increased risk of pre-diabetes. Consistent relationships have been found among non-diabetic U.S. adults, using data from NHANES III [38, 39], as well as Kuwait adults [40]. In addition, above results suggest that vitamin D deficiency has important effects on insulin resistance and impaired β-cell function, which are the two critical factors that drive the development of T2DM. Remarkably, in the following stratified analyses, we only observed the association between serum 25(OH)D and pre-diabetes among overweight or obese adults. Hypponen et al. [37] reported that obesity played an important role in the relation between serum 25(OH)D and glucose homeostasis. To some extent, our results were in agreement with the previous findings that overweight or obese adults had poorer vitamin D status [41, 42]. Nevertheless, another cross-sectional study among elder Brazilians indicated there was no association between 25(OH)D levels and pre-diabetes [43] due partly to their small sample size. Considering the higher prevalence of pre-diabetes among Chinese, a stage in the disease continuum where diabetes prevention has been shown to be effective [44, 45], identification and treatment of pre-diabetic individuals are therefore crucial.

In the meanwhile, our data indicated that vitamin D deficiency was fairly common in our study sample with an average age of 48.74 years (the prevalence of vitamin D deficiency or insufficiency was 79.8%), which was lower than another study focused on Chinese adults aged 50-70 years (93.6%, 2009) [46] perhaps owing to the fact that aging is associated with reduced capacity to produce vitamin D [20]. Notably, prevalence in this study was substantially higher than those observed among US population in 2009-2010 (64%) [47], which may lie in that fewer vitamin D fortified foods are available in China. Our results suggest that vitamin D deficiency might be universal in the general population in China and relevant measures might be taken to improve vitamin D status of Chinese adults.

Several limitations of our study merit consideration. Owing to the cross-sectional nature of the study design, a cause-effect relationship between serum 25(OH)D and glucose homeostasis cannot be inferred. The present study was completed within a relatively long period, from March to October, 2015, which may increase the seasonal variation in the biomarkers. However, data from the Ely Study (Cambridgeshire, U.K.) [13] and NHANES 2001-2006 [14] suggested that the associations between 25(OH)D and glucose homeostasis were independent of season. Finally, our analysis has excluded diabetic participants, many of whom were men due to the higher prevalence of diabetes in men than women among Chinese populations, generating an imbalanced sex proportion which could have potentially biased our results. Nonetheless, initial analysis indicated no interaction between gender and serum 25(OH)D, and we have adjusted gender in the multiple regression analyses. Furthermore, participants in the present analysis have been shown to be comparable to age-matched adults in the general population in Southwest China on sociodemographic and lifestyle characteristics [26].

Notwithstanding the limitations mentioned above, the present study had following strengths. Serum 25(OH)D allowed for objective measurement of vitamin D status rather than relying on self-reported vitamin D intake or sunlight exposure, which circumvented the recall bias. In addition, our analyses took into account many potential covariates that might confound the observed associations. Finally, we investigated participants aged 25-65 years with a range of quantitative indicators of glucose homeostasis (including FPG, fasting insulin, HbA_1c_ and HOMA2-IR), providing more comprehensive results than the previous studies.

## Conclusions

In conclusion, we have demonstrated inverse associations of serum 25(OH)D concentration with glucose homeostasis in Southwest Chinese adults without diabetes. Prospective studies and clinical trials are needed to confirm our findings.

## Additional files

Additional file 1: **Table S1.** The diagnosis of pre-diabetes (*n* = 1514). (DOCX 16 kb)

Additional file 2: **Table S2.** Multiple logistic regression odds ratio (OR) and 95% confidence interval (CI) for the association of tertiles of serum 25(OH)D (ng/ml) with pre-diabetes^1^ (DOCX 18 kb)

## Abbreviations

25(OH)D: 25-hydroxyvitamin D
BMI: Body mass index
CV: Coefficient of Variation
FPG: Fasting plasma glucose
HbA_1c_: Glycated hemoglobin
HOMA2-IR: The homeostatic model assessment 2-insulin resistance
MVPA: Moderate-to-vigorous physical activity
NHANES: National Health and Nutrition Examination Survey
OGTT: Oral glucose tolerance test
T2DM: Type 2 diabetes mellitus
VDRs: Vitamin D receptors
WC: Waist circumference
