# Supplementary material for: Higher serum 25(OH)D level is associated with decreased risk of impairment of glucose homeostasis: data from Southwest China
Source: BMC Endocr Disord. 2018 May 9;18:25. doi: 10.1186/s12902-018-0252-4 (PMC5941481; doi:10.1186/s12902-018-0252-4)
Supplement: Supplementary file 1 — Table S1. The diagnosis of pre-diabetes (n = 1514). (DOCX 16 kb) [file 12902_2018_252_MOESM1_ESM.docx]

**Table S1** The diagnosis of pre-diabetes (n=1514)

| Criterion^1^ | n (%) |
| --- | --- |
| An HbA_1c_ of 5.7%-6.4% | 602 (39.8) |
| FPG of 100-125 mg/dl (5.6-6.9mmol/L) | 762 (50.3) |
| HbA_1c_ of 5.7%–6.4% and FPG of 100-125 mg/dl (5.6-6.9mmol/L) | 377 (24.9) |
| total | 987 (65.2) |

^1^ It was defined using the updated classification and diagnosis of diabetes of American Diabetes Association [3].
