# Supplementary material for: Higher serum 25(OH)D level is associated with decreased risk of impairment of glucose homeostasis: data from Southwest China
Source: BMC Endocr Disord. 2018 May 9;18:25. doi: 10.1186/s12902-018-0252-4 (PMC5941481; doi:10.1186/s12902-018-0252-4)
Supplement: Supplementary file 2 — Table S2. Multiple logistic regression odds ratio (OR) and 95% confidence interval (CI) for the association of tertiles of serum 25(OH)D (ng/ml) with pre-diabetes1 (DOCX 18 kb) [file 12902_2018_252_MOESM2_ESM.docx]

**Table S2** Multiple logistic regression odds ratio (OR) and 95% confidence interval (CI) for the association of tertiles of serum 25(OH)D (ng/ml) with pre-diabetes^1^

|  | OR (95%CI) | | | *P* for trend |
| --- | --- | --- | --- | --- |
| Pre-diabetes^3^ (yes or no) | Tertile 1  12.6 (9.5, 15.1)^2^ | Tertile 2  20.6 (18.6, 23.1)^2^ | Tertile 3  31.9 (27.9, 38.9)^2^ |  |
| Total (n=1514) |  |  |  |  |
| Model A^4^ | 1.00 | 1.07 (0.75, 1.52) | 0.70 (0.48, 1.01) | 0.056 |
| Model B^5^ | 1.00 | 1.07 (0.74, 1.52) | 0.69 (0.47, 0.99) | 0.049 |
| Model C^6^ | 1.00 | 1.06 (0.74, 1.52) | 0.68 (0.47, 0.99) | 0.046 |
| BMI<25kg/m^2^ (n=1071) |  |  |  |  |
| Model A^4^ | 1.00 | 1.23 (0.82, 1.86) | 0.83 (0.53, 1.27) | 0.185 |
| Model B^5^ | 1.00 | 1.24 (0.82, 1.88) | 0.82 (0.53, 1.27) | 0.175 |
| BMI≥25kg/m^2^ (n=443) |  |  |  |  |
| Model A^4^ | 1.00 | 0.60 (0.26, 1.33) | 0.36 (0.16, 0.80) | 0.049 |
| Model B^5^ | 1.00 | 0.60 (0.26, 1.34) | 0.36 (0.16, 0.79) | 0.048 |

^1^ Values are odds ratio and 95% confidence interval. Linear trends (*P* for trend) were obtained with vitamin D concentrations as continuous variables;

^2^ Values are median (25th percentile, 75th percentile) of in tertiles of Vitamin D (ng/ml);

^3^ Using the Classification and Diagnosis of diabetes of American Diabetes Association to classify pre-diabetes [3];

^4^ Model A: adjusted for age, gender, average personal income per month, smoking status and season of blood drawn;

^5^ Model B: additionally adjusted for physical activity and energy intake;

^6^ Model C: additionally adjusted for waist circumference.
